# Feeding ecology of monk sakis (*Pithecia monachus*) in a seasonally flooded forest in western Amazonia

**DOI:** 10.1007/s10329-023-01074-9

**Published:** 2023-06-21

**Authors:** Malika Gottstein, Abigail Lauren Morris, Katrin Heer, Eckhard W. Heymann

**Affiliations:** 1 Faculty of Environment and Natural Resources, Eva Mayr-Stihl Professorship for Forest Genetics, Albert-Ludwigs-Universität Freiburg, Freiburg im Breisgau, Germany; 2 Animal Behavior Graduate Group, University of California, Davis, USA; 3 Deutsches Primatenzentrum, Leibniz-Institut für Primatenforschung, Göttingen, Germany

**Keywords:** Frugivory, Diet, Primates, Platyrrhines, Feeding plants, Arthropod consumption

## Abstract

Sakis (genus *Pithecia*) are frugivorous primates with a preference for seeds that complete their diet with leaves and insects. Fruit pulp and seeds are known to have different nutritional characteristics that change during the process of ripening. The consumption of seeds can be an adaptation to changes in resource availability, as unripe seeds are a more steadily available resource than ripe pulp or young leaves. Here, we present the first study of the feeding ecology of monk sakis (*Pithecia monachus*). We investigated dietary composition and identified important feeding plants in a seasonally flooded forest within the Área de Conservación Regional Comunal Tamshiyacu–Tahuayo in Peruvian Amazonia. Throughout 20 months, we followed groups of monk sakis by foot and canoe and recorded 459 feeding events. Seeds were the most frequently consumed food item (49%), followed by pulp (mesocarp, pericarp or aril; 25%) and arthropods (22%). Leaves, bark, and flowers were ingested only sporadically. The importance of ripe seeds and arthropods in the diet of the monk sakis differed from other studies: we recorded the consumption of mostly ripe seeds and the share of arthropods was relatively high.

## Introduction

In tropical forests, fruits are an important food resource for many vertebrate species (Fleming et al. 1978). Fruits comprise several nutrient-rich components with different characteristics that change with the developmental stage and can differ between taxa (Janzen 1983). The pulp can be formed by a sugary pericarp or mesocarp, or a lipid-rich aril (Janzen 1983; Norconk 2021). Seeds additionally contain a high proportion of protein, but are usually physically and chemically protected to prevent seed predation (Janzen 1976; Norconk and Veres 2011). One vertebrate group that particularly depends on fruit as a resource are primates (Richard 1985). All South American primates (Platyrrhini) rely on fruit as part of their diet, although the proportion in the diet and which part of a fruit is used varies greatly (Rosenberger 2020).

The feeding ecology of *Pithecia* spp. is understudied, since sakis are difficult to habituate and very shy (Pinto et al. 2013). Of the 16 species considered by Marsh (2014), information on feeding ecology is available for only eight, and often restricted to short study periods or few observations. The majority of studies has been conducted on *P. pithecia* in the Guianan region. Only two studies have been conducted on sakis in seasonally flooded habitats, namely on *P. rylandsi*^1^ (Palminteri et al. 2012) and *P. isabela*^2^ (Soini 1987).

Sakis are frugivores with a strong preference for seeds that made up 53–70% of their diet in previous studies (Norconk and Conklin-Brittain 2004: 63%, *P. pithecia*; Palminteri et al. 2012: 70%, *P. rylandsi*; Peres 1993: 53%, *P. albicans*). Seeds are usually masticated and sakis therefore act as “seed predators” (Ledogar et al. 2013; Norconk 2021). They have a highly specialized dental morphology to break open hard-shelled fruits and masticate seeds before swallowing (Kay et al. 2013; Kinzey and Norconk 1990; Norconk and Veres 2011). The consumption of seeds has been described as an adaptation to variation in fruit availability, as they are a more steadily available resource than ripe pulp (Norconk 1996; Palminteri et al. 2012). Like pulp, seeds change their chemical composition during the process of ripening of the fruit (Norconk and Conklin-Brittain 2004). For example, lipid levels increase and tannin levels decrease during seed ripening in certain plants consumed by sakis in Venezuela (Kinzey and Norconk 1993). The authors of previous studies found that >  95% of the seeds consumed by various saki species were unripe (Norconk 1996: 100%, *P. pithecia*; Oliveira et al. 1985: 100%, *P. chrysocephala*;^3^ Palminteri et al. 2012: > 99%, *P. rylandsi*; Peres 1993: > 98%, *P. albicans*).

Sakis complement their diet with pulp, leaves, and insects (Happel 1982; Izawa 1975; Kinzey 1992; Ledogar et al. 2013; Norconk 1996; Norconk and Setz 2013; Oliveira et al. 1985; Peres 1993; Soini 1987). When consuming pulp, sakis prefer ripe mesocarp and arils (Charpentier et al. 2015; Norconk 1996; Peres 1993). Arthropods are not always listed as part of the diet or only represent a share of < 10% of the ingested food (Cunningham and Janson 2006: < 10%, *P. pithecia*; Kinzey and Norconk 1993: < 6%, *P. pithecia*; Peres 1993: < 1%, *P. albicans*). However, insect consumption can help to nutritionally complement the frugivorous diet (Rothman et al. 2014; Urbani et al. 2019).

Sakis are distributed throughout the Amazon basin and inhabit different types of forest, including high ground terra firme forest and seasonally flooded forest, white-water várzea, and black-water igapó (Marsh et al. 2018; Palminteri and Peres 2012). The former has usually an increased productivity due to the nutrient deposition during the annual flooding (Junk 1997; Melack and Forsberg 2001), although this is less distinguishable in western Amazonia (Prance 1979). Sakis use a variety of plant taxa, including many that are not as important in the diet of other platyrrhine species (Boyle et al. 2016; Norconk 2021). Plant families such as Moraceae, Fabaceae, Chrysobalanceae, Sapotaceae, Annonaceae, and Lecythidaceae have been repeatedly reported to be part of their diet (Charpentier et al. 2015; Happel 1982; Norconk 1996; Peres 1993; Setz 1993). Pitheciine biomass is positively correlated with the abundance of *Eschweilera* trees (Lecytidaceae) (Stevenson 2001). *Inga* (Fabaceae), *Brosimum* (Moraceae), and *Pouteria* (Sapotaceae) are particularly important in pitheciine diets across different habitats (Boyle et al. 2016).

Here, we present the first study on the feeding ecology of monk sakis (*P. monachus*). We determine the monk sakis’ dietary composition and identify important feeding plants. Based on previous studies on saki feeding ecology, we expected unripe seeds to be the most consumed food of monk sakis, followed by ripe fruit pulp. We anticipated that arthropods will be consumed occasionally, but will not make up a large proportion of the diet. Since *Eschweilera*, *Pouteria*, and *Inga* are present in the flooded forests and were reported to be important food items for other saki species, we expected these to also be included in the monk saki diet at our study site.

## Material and methods

### Study site

The Área de Conservación Regional Comunal Tamshiyacu-Tahuayo (ACRCTT) is located in northern Peruvian Amazonia in the department of Loreto. It was first established in 1991 as a Reserva Comunal, but was given added protections and expanded to its current size of 420,000 ha in 2009 (Gobierno Regional de Loreto 2009). The ACRCTT is known for its high biodiversity and is home to 13 primate species (Heymann and Aquino 1994; Valqui 2001). Before its designation as a protected area, primates, including saki monkeys, were hunted in the region (Bodmer 1995; Newing and Bodmer 2003). The area consists mostly of nonflooded terra firme habitat, but also contains seasonally flooded forests (Gobierno Regional de Loreto 2009). Mean monthly temperatures range from 25 to 27 °C and annual rainfall in the Tahuayo River Basin is ca. 3000 mm (Myster 2015).

**Fig. 1:**
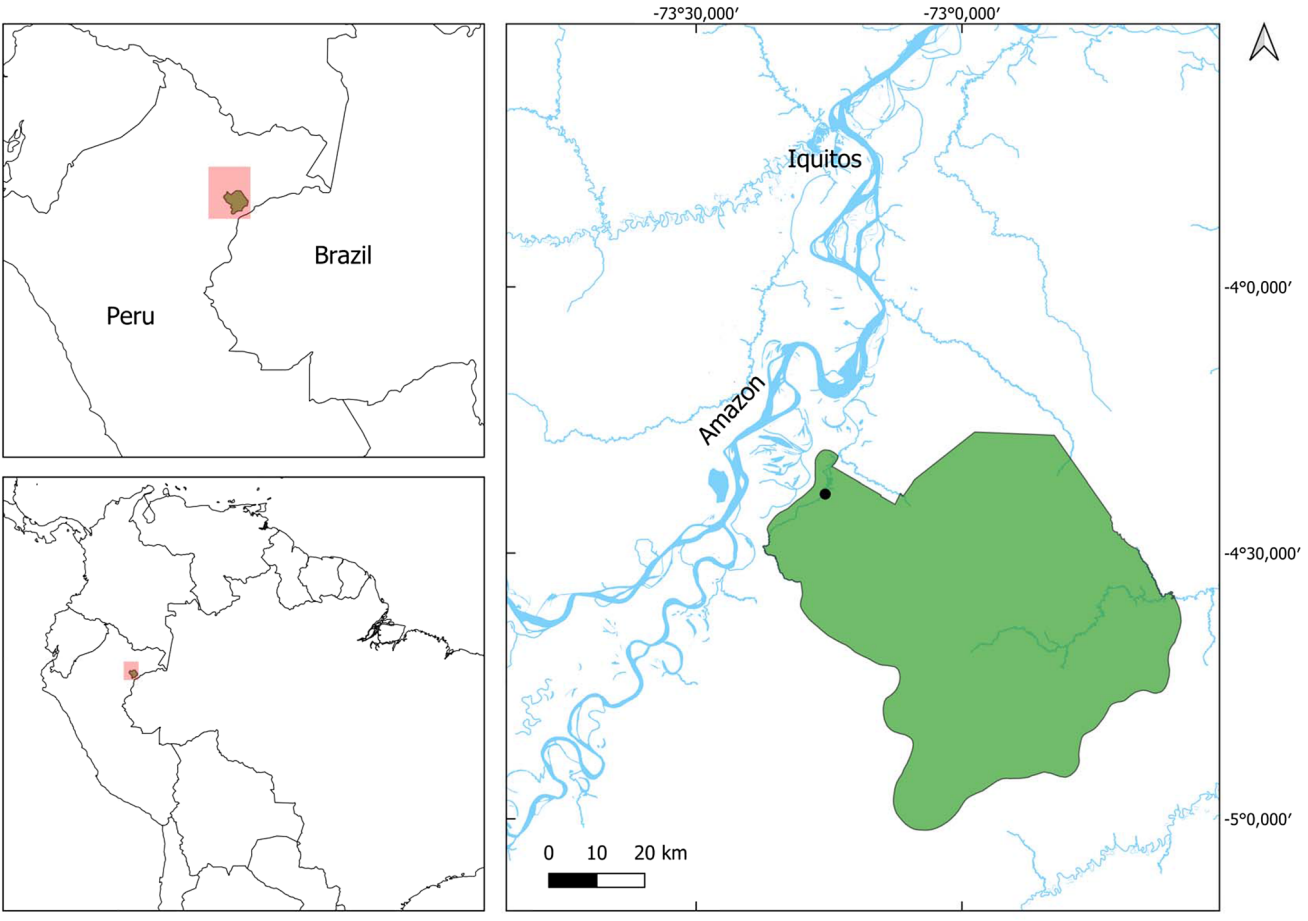
Map showing the location of the Amazon Research Center (ARC; ●) within the Área de Conservación Regional Comunal Tamshiyacu-Tahuayo (ACRCTT; green polygon). The river in the lower right corner of the map (Rio Yavarí) marks the border with Brazil

We collected the data around the Amazon Research Center (ARC) (4°22′23′′–4°24′16′′S 73°14′45′′–73°16′36′′W, Fig. 1). The ARC is located in the floodplain of the Tahuayo River, a tributary of the Amazon River, and run by the ecotourism company Amazonia Expeditions (www.perujungle.com). The Tahuayo is primarily an acidic, black-water river with a low nutrient content (Myster 2015). River water levels increase around mid-November, lower ecosystems begin to flood by the end of January, and widespread flooding is experienced from late March through to the end of May (“flood season”) (Gobierno Regional de Loreto 2009; Ronchail et al. 2018). From June to October, river levels are low (“dry season”) (Gobierno Regional de Loreto 2009). When referring to the “dry season” in our study area, we address the months with less rainfall and lower river levels, although precipitation levels are high throughout the year (Kelly et al. 2014).

### Study species and groups

Our study species, the monk saki (*P. monachus*), is distributed in Peru and Brazil, in the interfluvium area between the Amazon/Solimões, lower-to-middle Ucayali, and lower Juruá rivers (Marsh et al. 2018). Their general shy behavior and inconspicuous coloration pose significant challenges to conducting field observations of *P. monachus* (Bartecki and Heymann 1987; Pinto et al. 2013). In addition to their position as a prey species for a variety of predators, these monkeys remain cryptic due to their history as a hunted population in this region (Marsh 2014).

We followed at least 12 groups of monk sakis inside the ACRCTT and in its buffer zone. Group size varied between two and seven individuals, most groups consisted of five individuals (median = mean = 5). Each group was composed of at least one adult male and one adult female (easily distinguished by their sexually dichromatic coloration), and some groups had more than two adult individuals. Juveniles were present in 11 groups. Infants were present in two groups during the study.

We searched for and followed groups of saki monkeys in the ACRCTT from July 2019 to July 2020 and from August 2021 to May 2022. The saki groups were not habituated and were typically very shy (Jackson 2016; Lehtonen 2017; Stenzel 2017). We left the ARC in the early morning, between 5 and 7 am. We alternately searched downstream (north of the ARC), upstream (southwest), and inland away from the river (southeast). We spent 931 h searching for sakis in the dry season and 1463 h in the flood season (Table 1). During the dry season, we used a canoe to move upstream or downstream and then followed by foot after locating a group of sakis. Searching inland was done by foot during the dry season. During the flood season, we used a small boat to move on the river and changed into a canoe after locating a group. Searching inland was done by canoe during the flood season. We located the sakis either visually, generally by witnessing movement, or via hearing their calls. Upon locating a group, we followed them for as long as possible. We defined contact hours as the amount of time that we were in visual contact with sakis or that we knew where the sakis were, e.g., hiding in a tree. In the latter case, we continuously watched the hiding place, to detect movement or observe feeding. If possible, we followed a group of sakis until nightfall and came back before sunrise the next morning. The total contact hours with each group varied between 4 and 90 h (total effort = 614 contact hours).

**Table 1:**
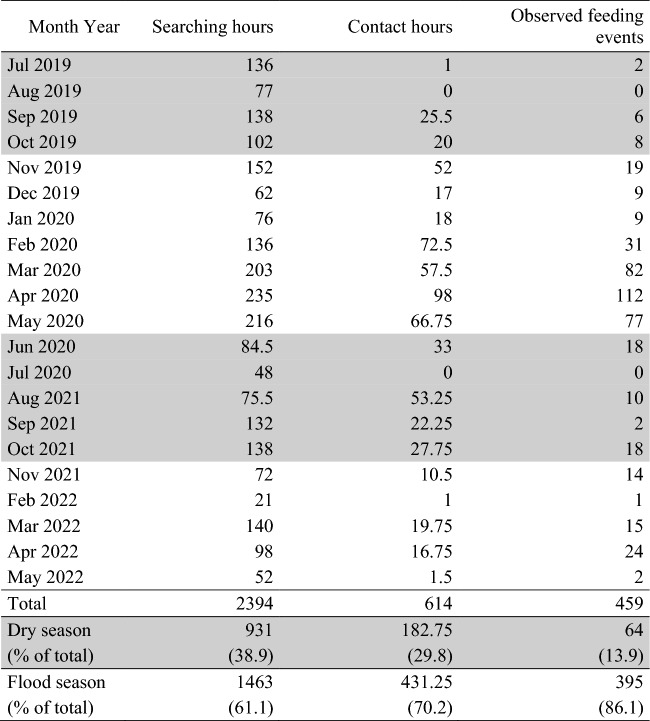
Hours spent searching for sakis, including contact hours and observed feeding events for each month of the study period (July 2019 to July 2020 and August 2021 to May 2022)

Months and numbers in gray represent the dry season

### Feeding observations

We used behavioral sampling (Martin and Bateson 2007) to collect data on feeding with the help of binoculars. When we observed feeding, we noted the date, time, and number of sakis. We defined a feeding event as one individual feeding on a specific food item, independent of the time they spent feeding or the amount of food ingested. For example, if we saw a group of five sakis feeding on seeds in a tree, we recorded five feeding events, regardless of the number of seeds consumed by each individual. Accordingly, if we saw one saki feeding on several ants from the same branch, we recorded one feeding event. If a saki fed on plant parts, we recorded the Global Positioning System (GPS) location of the respective plant, marked it with tape for future recognition, and collected fruit, leaf, and bark samples for identification by botanists of the Herbarium AMAZ of the Universidad Nacional de la Amazonía Peruana in Iquitos.

**Fig. 2:**
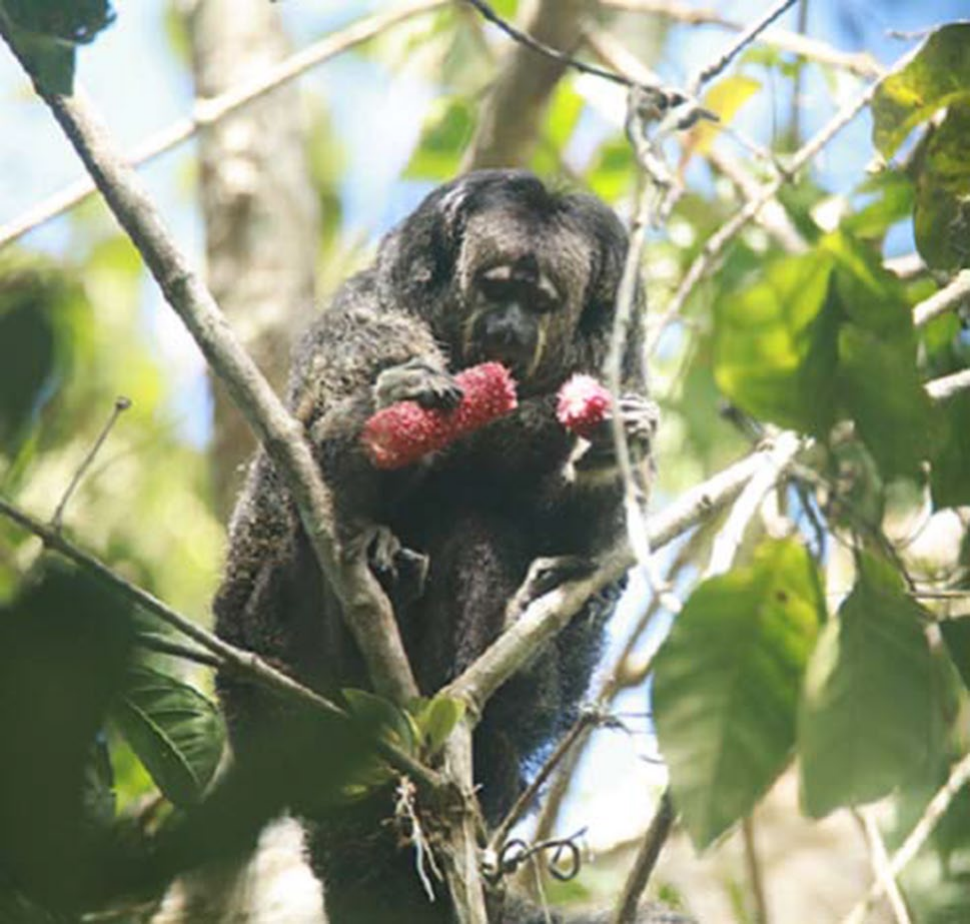
Monk saki feeding on *Anthurium smithii* (Araceae) (Photo: M. Gottstein)

We classified the type of food eaten as seed, mesocarp, pericarp, aril, leaf, bark, flower, or arthropod. For each feeding event on fruit parts, we assigned a category of ripeness: ripe, unripe, ripe + unripe (if both ripe and unripe fruits were consumed during the same feeding event), dry and NA (if we were unable to assign a category). We collected the fruits that the sakis dropped to inspect which part had been consumed and compared with intact fruits on the ground. We stored the fruits in 70% ethanol and labeled the vials with the number we assigned to the respective feeding tree. We determined ripeness preliminarily by looking at the size and color of the fruit and by opening an intact fruit of similar size to assess the stage of seed and pulp development. If possible, we took photos of the feeding plant and event (Fig. 2). Toward the end of our field study, we corrected some of our assignments of ripeness by comparing the stored fruit samples with ripe fruits collected throughout the year. If fruit pulp was consumed, we specified which part of the fruit becomes fleshy and constitutes the pulp (mesocarp, pericarp, or aril) following the genus-wise classification of pulp by Cornejo and Janovec (2010) and van Roosmalen (1985). For arthropods, we recorded the substrate from which the item was picked as stem, branch, leaf, epiphyte, or out of the air. If it was visible, we noted the class, order, or family of consumed arthropods; a higher taxonomic resolution was not possible.

## Results

### Diet composition

Seeds were the most frequently consumed food item (49%, *n* = 226), followed by arthropods (22%, *n* = 99), mesocarp or pericarp (14%, *n* = 64), mesocarp or pericarp plus seed (7%, *n* = 33), aril plus seed (4%, *n* = 20), leaves (2%, *n* = 10), bark (1%, *n* = 5), and flowers (< 1%, *n* = 2). The majority of feeding events on fruit items (seed, mesocarp, pericarp, and aril) came from ripe fruits (Table 2).

**Table 2:** Number of feeding events on fruit items (seed, mesocarp, pericarp, aril) and their ripeness

	Ripe	Unripe	Ripe + unripe	Dry	NA
Seed	148 (26, 122)	50 (13, 37)	17 (0, 17)	8 (0, 8)	3 (2, 1)
Mesocarp/pericarp	50 (17, 33)	7 (0, 7)	0 (0, 0)	0 (0, 0)	5 (1, 4)
Mesocarp/pericarp + seed	28 (0, 28)	1 (0, 1)	4 (0, 4)	0 (0, 0)	0 (0, 0)
Aril + seed	19 (0, 19)	1 (0, 1)	0 (0, 0)	0 (0, 0)	0 (0, 0)
Total	245	59	21	8	8

Number in parentheses indicate the number of feeding events in the dry season (first number) and the flood season (second number)

### Food items

We observed the monk sakis feeding on 212 plants, of which 108 could be identified to genus and 72 to species level. Food plants came from 29 plant families (Table 2). The plant items consumed by the sakis were from species that grow as tree (*n* = 49), vine (*n* = 14), shrub (*n* = 5), palm (*n* = 1), or epiphyte (*n* = 1). Most feeding events concerned plants from *Eschweilera* (*n* = 53, Lecythidaceae) and *Pouteria* (*n* = 30, Sapotaceae). We were not able to identify the species of consumed dry seeds, bark, or flowers (Table 3).

**Table 3:** Plant species and items consumed by monk sakis in July 2019–July 2020 and Aug 2021–May 2022

Family (no. of feeding events)	Species	Jan	Feb	Mar	Apr	May	Jun	Jul	Aug	Sep	Oct	Nov	Dec	No. of feeding events per species	Fruit type
Lecythidaceae (54)	*Eschweilera* sp.			rS					rS	uS, rS	uS, rS	uS, rS	rS	33	Pyxidium^a^
	*Eschweilera parvifolia*		uS									rS	rS	13	Pyxidium^a^
	*Eschweilera grandiflora*		rS								rS	rS		4	Pyxidium^a^
	*Eschweilera coriacea*		rS											2	Pyxidium^a^
	*Couratari oligantha*		rS											1	Pyxidium^a^
	*Eschweilera albiflora*											rS		1	Pyxidium^a^
Sapotaceae (33)	*Pouteria cuspidata*				uS	uS, rS								9	Berry^a^
	*Pouteria cladantha*				uS									7	Berry^a^
	*Pouteria boliviana*			rP										5	Berry^a^
	*Pouteria franciscana*		rP											5	Berry^a^
	*Pouteria guianensis*		rP, rS	rS	uS									4	Berry^a^
	*Chrysophyllum manaosense*				rS									3	Berry^a^
Myrtaceae (23)	*Psidium densicomum*	rS				uP, uS, rS								9	Berry^a^
	*Psidium* sp.					uS								5	Berry^a^
	*Eugenia* sp. 1			rS		rS								4	Berry^a^
	*Myrciaria* sp. 1			rS										2	Berry^a^
	*Myrciaria* sp. 2			rS										2	Berry^a^
	*Eugenia* sp. 2					uS								1	Berry^a^
Myristicaceae (18)	*Virola pavonis*		rA, rS	rA, rS								rA, rS		9	Capsule^a^
	*Virola marlenei*			rA, rS										4	Capsule^a^
	*Iryanthera grandis*			rA, rS										2	Capsule^a^
	*Iryanthera lancifolia*											uS		1	Capsule^a^
	*Iryanthera macrophylla*				rA, rS									1	Capsule^a^
	*Virola calophylla*											uS		1	Capsule^a^
Arecaceae (14)	*Mauritia flexuosa*		rM				rM		rM					14	Drupe^a^
Passifloraceae (10)	*Passiflora involucrata*					uS								4	Berry^a^
	*Dilkea acuminata*					rM, rS								2	Berry^a^
	*Passiflora* sp.				rM, rS									2	Berry^a^
	*Dilkea retusa*			uS										1	Berry^a^
	*Passiflora nitida*			uS										1	Berry^a^
Fabaceae (9)	*Inga brachyrhachis*		rA, rS											5	Legume^a^
	*Swartzia simplex*				rA, rS									3	Legume^a^
	*Swartzia benthamiana*					rA, rS								1	Legume^a^
Clusiaceae (8)	*Clusia amazonica*			uA, uS										4	Capsule^a^
	*Clusia* sp.				rA, uA, rS, uS									2	Capsule^a^
	*Clusia viscida*								rS					1	Capsule^a^
	*Havetiopsis flexilis*				L									1	
Olacaceae (8)	*Dulacia candida*			rS										4	Drupe^b^
	*Tetrastylidium peruvianum*					uS								3	
	*Heisteria scandens*											uS		1	Drupe^b^
Sterculiaceae (7)	*Theobroma subincanum*			rP, rS										6	Berry-like capsule^a^
	*Theobroma obovatum*											uP		1	Berry-like capsule^a^
Apocynaceae (6)	*Macoubea guianensis*				rM									3	Berry^a^
	*Rhigospira quadrangularis*				rM, rS									2	
	*Ambelania occidentalis*				rS									1	Berry^a^
Sapindaceae (6)	*Paullinia alata*			rS	rS									5	Capsule^a^
	*Matayba arborescens*								rS					1	Capsule^a^
Chrysobalanaceae (5)	*Couepia macrophylla*												rM,rS	2	Drupe^a^
	*Licania* sp.	rM, rS												2	Drupe^a^
	*Couepia subcordata*				rM, rS									1	Drupe^a^
Euphorbiaceae	*Mabea nitida*			rS, uS										4	Capsule^a^
	*Mabea speciosa*			rS, uS										1	Capsule^a^
Loganiaceae	*Strychnos guianensis*					rM, rS								3	Berry^a^
	*Strychnos* sp.					rM, rS								1	Berry^a^
Convolvulaceae	*Maripa peruviana*						rS							2	Drupe^a^
	*Ipomoea* sp.					rS								1	Capsule^a^
Araceae	*Anthurium smithii*					rM, rS								2	Berry^b^
Bixaceae	*Bixa arborea*	rS												2	Capsule^a^
Humiriaceae	*Sacoglottis mattogrossensis*			rS										1	Drupe^b^
	*Vantanea* sp.			rS										1	Drupe^b^
Malvaceae	*Pachira brevipes*			rS	rS									2	Capsule^a^
Marcgraviaceae	*Norantea* sp.			rS										2	Capsule^a^
Violaceae	*Leonia glycycarpa*						rS							2	Berry^a^
Caryocaraceae	*Anthodiscus pilosus*				rS									1	Drupe^a^
Celastraceae	*Salacia impressifolia*										uS			1	Drupe^b^
Dilleniaceae	*Doliocarpus dentatus*				L									1	
Elaeocarpaceae	*Sloanea durissima*	rS												1	Capsule^a^
Loranthaceae	*Psittacanthus calcaratus*				rM, rS									1	Drupe^a^
Primulaceae	*Cybianthus nestorii*				rS									1	Drupe^b^
Rubiaceae	*Alibertia* sp.		rM											1	Berry^a^

*A* aril, *L* leaf, *M* mesocarp, *P* pericarp, *S* seed, *r* ripe, *u* unripe. Classification of fruit types follows Cornejo and Janovec (2010) (a) and van Roosmalen (1985) (b). Families are listed in descending order of the number of feeding events (figures in brackets after family name); within families, species are also listed in descending order of the number of feeding events

The sakis fed on arthropods picked up from branches (*n* = 27), leaves (*n* = 24), epiphytes (*n* = 11), the air (*n* = 5), or from the main stem of a tree (*n* = 3). For 29 feeding events on arthropods, we did not see the substrate from which the consumed arthropod was taken. We observed the consumption of termites (*n* = 5), ants (*n* = 4), katydids (*n* = 4), small spiders (*n* = 2), and butterfly (*n* = 1). We were not able to classify the majority of the remaining arthropods (*n* = 83)

## Discussion

Observing sakis is generally difficult, and the groups encountered at our study site were shy. We spent many days in the field without seeing the sakis. Especially during the dry season, it was very difficult to observe feeding, since we made noise walking on the forest floor and the sakis hid for the rest of the day when perceiving us. The number of observations is therefore relatively low for some months. Nevertheless, we were able to contribute knowledge on saki feeding ecology that can help us discover species- and habitat-specific differences.

The high proportions of seed and pulp, respectively, in the diet support the classification of monk sakis as frugivores with an emphasis on seeds. However, we could only partially confirm our expectation that unripe seeds would be the most important food source for monk sakis, since most consumed seeds were already ripe. We could confirm the expectation that ripe fruit pulp would be the second most important resource for monk sakis.

Ripe seeds can be nutritionally different from unripe seeds. For example, ripe seeds had higher lipids and free simple sugar, and lower crude protein than unripe seeds consumed by sakis in Venezuela (Norconk and Conklin-Brittain 2004). Other studies that collected data throughout different seasons found changes in the dietary composition, such as changing proportion of ripe fruit items, increased use of certain plant species, or consumption of arthropods and leaves (Kinzey and Norconk 1993; Norconk 1996; Palminteri et al. 2012; Soini 1987). These changes do not appear to be generalizable across habitats and regions. We observed the majority of feeding events (86%) during the flood season. Although we did not conduct a phenological survey, it is likely that the availability of ripe and unripe seeds changes throughout the year, with implications for saki feeding ecology. Because of the difference in the number of observations during the wet and dry seasons, we refrained from examining possible seasonal patterns in dietary composition and use of feeding plants. However, it is possible that the high proportion of ripe seeds in the diet is due to seasonal variation in resource availability.

Contrary to our expectations, we found that arthropods were consumed regularly and accounted for the third-highest number of feeding events (22%). One possible explanation for this difference with other studies might be that we used a different way of quantification of feeding observations. While we used the number of feeding events to measure the importance of a resource in the saki diet, most other studies used feeding time as a measurement (e.g., Cunningham and Janson 2006; Kinzey and Norconk 1993; Peres 1993). Since the consumption of arthropods likely takes less time than, for example, opening a fruit to feed on the seed, the relative importance of arthropods in the sakis’ diet might be much higher when determined based on event instead of feeding time. Nevertheless, we clearly showed that arthropods are an important resource for monk sakis. Another possible explanation is the time period and habitat our study took place in. In seasonally flooded forests, arthropods might be more readily available for sakis in the flood season, since some taxa move into higher forest strata during flooding (Adis 1992; Irmler 1979; Souza et al. 2020). The authors of studies on arthropod diversity from saki feces suggest that the importance of arthropods in their diet might be underestimated due to the difficulties of observing sakis in the wild (Jesus et al. 2022; Pickett et al. 2012).

We identified 70 species of feeding plants from 29 families. The most used plant family was Lecythidaceae, with seeds from *Eschweilera* being the most consumed. *Eschweilera* has been shown to be of special importance to sakis (Stevenson 2001) and its seeds were reported to be the most consumed by *P. isabela* in a seasonally flooded forest in western Amazonia (Soini 1987). The increased use of *Mauritia flexuosa* during some months was also described by Palminteri et al. (2012). Not all plant genera found to be of special importance for pitheciines in a meta study (Boyle et al. 2016) were recorded in our observations: seeds and pericarp of *Pouteria* were the second most consumed food items, but *Inga* was less important (*n* = 5), and *Brosimum* not recorded at all. Also, the plant families Moraceae and Annonaceae that were reported as important in previous studies, were not present in our observations. However, we were not able to identify all plant items the sakis fed on. The high diversity of plants at our study site and supra-annual patterns of fruiting make it likely that we only recorded a fraction of the plants actually used by monk sakis.

Altogether, we found monk sakis in a seasonally flooded forest in western Amazonia to have a similar feeding ecology to other saki species in distinct habitats. However, we also found some differences. Seeds were more often consumed in ripe state and arthropods were more important than we expected based on the literature. Monk sakis might have a slightly different feeding strategy than other saki species, although we suspect that these dietary differences result mainly from environmental conditions. It would be instrumental to study adjacent monk saki populations that are restricted to nonflooded forest. Research is needed to better understand saki feeding ecology, especially in western Amazonia.

## Data Availability

Not applicable.

## Footnotes

1 Previously *P. irrorata*; see Marsh (2014) for taxonomic change.

2 Previously *P. monachus*; see Marsh (2014) for taxonomic change.

3 Previously *P. pithecia*; see Marsh (2014) for taxonomic change.
